# Supplementary material for: Frictional fluid instabilities shaped by viscous forces
Source: Nat Commun. 2023 May 26;14:3044. doi: 10.1038/s41467-023-38648-6 (PMC10220059; doi:10.1038/s41467-023-38648-6)
Supplement: Supplementary file 2 — Description of Additional Supplementary Files [file 41467_2023_38648_MOESM2_ESM.docx]

**Description of Additional Supplementary Files**

File Name: Supplementary Movie 1

Experiment and simulation of viscously stable invasion with growth of a single frictional finger. Viscosity of invading fluid $\eta_{\text{inv}}=1$ mPa.s, injection rate *Q* = 1 ml.min^-1^, filling fraction *ϕ*  = 0.5, Hele-Shaw cell gap thickness *b* = 0.9 mm, disc diameter 26.8 cm (scale). The real time duration of the movie is 75 s.

File Name: Supplementary Movie 2

Experiment and simulation of viscously stable invasion with simultaneous growth of multiple fingers. $\eta_{\text{inv}}=1$ mPa.s,  *Q* = 30 ml.min^-1^, *ϕ* = 0.5, *b* = 0.9 mm, disc diameter 26.8 cm (scale). The movie is in real time.

File Name: Supplementary Movie 3

Experiment and simulation of viscously stable invasion with formation of radial spoke pattern. $\eta_{\text{inv}}=1414$ mPa.s,  *Q* = 10 ml.min^-1^, *ϕ* = 0.5, *b* = 0.9 mm, disc diameter 26.8 cm (scale). The real time duration of the movie is 109 s.
